# Symptom Duration as a Predictor of Neurological Recovery After Decompression Surgery for Degenerative Cervical Myelopathy

**DOI:** 10.7759/cureus.97729

**Published:** 2025-11-25

**Authors:** Yazan Noufal, Felix Schmitz, Marcus Richter, Philipp Hartung, Philipp Drees, Yama Afghanyar, Martin Naisan

**Affiliations:** 1 Center of Orthopaedics, Spine and Trauma, St. Josefs-Hospital Wiesbaden, Wiesbaden, DEU; 2 Department of Orthopaedics and Traumatology, University Medical Center, Johannes Gutenberg University, Mainz, DEU

**Keywords:** degenerative cervical myelopathy, joa score, neurological recovery, prognostic factors, symptom duration

## Abstract

Background

The impact of symptom duration on postoperative recovery in degenerative cervical myelopathy (DCM) remains controversial. While earlier decompression is often recommended, patient outcomes are likely influenced by a combination of factors. This study aimed to evaluate whether the duration of symptoms significantly affects neurological recovery.

Methods

We performed a retrospective analysis of 142 consecutive patients (mean age 66.2 years, 63% male) who underwent anterior or posterior cervical decompression. Baseline variables included age, sex, body mass index (BMI), American Society of Anesthesiologists (ASA) status, number of stenotic segments, surgical approach, preoperative Japanese Orthopaedic Association (JOA) score, and duration of symptoms. Neurological function was assessed by JOA score preoperatively and at three and 12 months postoperatively. Multivariate linear regression identified independent predictors of continuous JOA change, while multivariate logistic regression assessed factors associated with achieving a clinically meaningful gain (≥1.5 points in JOA score).

Results

Overall, JOA scores improved by 1.16 ± 2.1 points (8.9%; p < 0.001) at three months and by 1.60 ± 2.2 points (12.2%; p < 0.001) at 12 months. Patients with severe myelopathy (JOA < 12) demonstrated the greatest gains (+1.99 at three months; +4.58 at 12 months; both p < 0.01), whereas those with mild symptoms showed minimal change. In multivariate analysis, a lower baseline JOA score was the sole predictor of greater continuous improvement at three months (p < 0.001) and remained significant at 12 months alongside ASA class (p = 0.017) and BMI (p = 0.027). Symptom duration did not predict the magnitude of continuous change. However, each additional month of preoperative symptoms decreased the odds of achieving a ≥1.5‐point gain at three months by 0.7% (p = 0.045). By 12 months, only patient sex (male vs. female; OR: 0.81; p = 0.046) independently predicted clinically meaningful recovery.

Conclusion

Baseline neurological impairment is the strongest determinant of postoperative improvement in DCM, with systemic health also influencing long‐term gains. Although longer symptom duration modestly reduces the likelihood of early clinically meaningful recovery, substantial improvement remains achievable after delay. These findings reinforce the importance of early diagnosis and timely surgical referral.

## Introduction

Degenerative cervical myelopathy (DCM) is the most common cause of spinal cord dysfunction in adults, resulting from progressive degenerative changes such as disc herniation, osteophyte formation, and ligamentous hypertrophy that lead to spinal cord compression [1]. Surgical decompression remains the treatment of choice to halt disease progression and to promote neurological recovery [2]. However, postoperative outcomes vary considerably among patients, and identifying reliable predictors of recovery remains a key challenge in clinical decision-making.

One factor that has been frequently discussed is the duration of preoperative symptoms [3-6]. Prolonged spinal cord compression may lead to chronic ischemia, axonal loss, and demyelination [7,8]. However, the prognostic impact of symptom duration remains controversial: while multiple cohort studies have demonstrated that delays beyond four months are associated with significantly poorer functional outcomes after decompression, other series have found no meaningful difference in the magnitude of postoperative improvement between early and delayed surgery, suggesting that substantial recovery may still be attainable even after prolonged symptom duration.

The primary objective of this study was to determine whether the duration of preoperative symptoms independently predicts neurological improvement after decompression surgery for DCM. We hypothesized that longer preoperative symptom duration would be associated with a lower likelihood of early- and mid-term neurological improvement.

Secondary objectives were to evaluate (1) the association between baseline Japanese Orthopaedic Association (JOA) score and recovery and (2) the influence of additional clinical factors such as American Society of Anesthesiologists (ASA) class, body mass index (BMI), age, sex, and surgical approach.

## Materials and methods

Ethical approval

The study was conducted according to the guidelines of the Declaration of Helsinki and approved by the Ethics Committee of the State Medical Association of Hesse (Ethikkommission Landesärztekammer Hessen) (approval number: 2025-3982-evBO). As this study is retrospective in nature, the need for informed consent was waived by the ethics committee.

Data collection

We collected data from 142 patients treated at our clinic, Center of Orthopaedics, Spine and Trauma, St. Josefs-Hospital Wiesbaden, Wiesbaden, Germany, between 2020 and 2025 who underwent surgical treatment for DCM. The data were extracted from the intrahospital information system (ORBIS, Agfa Healthcare GmbH, Bonn, Germany). 

Patients were eligible for inclusion if they had a diagnosis of DCM confirmed by both clinical signs and cervical MRI findings. Additional inclusion criteria were an age of 18 years or older, surgical treatment via anterior or posterior approach, and the availability of both preoperative and postoperative functional assessments using the JOA score. Patients were excluded if the myelopathy was due to trauma or neoplastic, infectious, or inflammatory causes. Cases in which revision surgery constituted the index procedure were also excluded from the analysis.

Collected variables included age, sex, BMI, ASA status classification, symptom duration, and preoperative functional scores (JOA). Surgical data included the number of operated levels, surgical approach, operative time, and intraoperative complications.

Symptom duration was patient-reported and, when possible, corroborated with clinical documentation from the first neurological evaluation or initial outpatient presentation.

Outcome measures

The primary outcome of this study was neurological improvement, defined as the change in JOA score between baseline and the follow-up assessments at three months and 12 months postoperatively. 

The JOA score is a validated clinical scale widely used to assess the severity of DCM. It ranges from 0 to 17 points, with higher scores indicating better neurological function [9]. The JOA score assesses upper and lower extremity motor and sensory function as well as bladder control and has been utilized in previous studies.

Since the JOA score is an established and non-proprietary measure, no specific permission was required for its use in this research.

A score of 15-17 is considered mild, 12-14 is moderate, and 0-11 is severe. Preoperative and three-month postoperative JOA scores were available for all patients, and 12-month follow-up data were available for 84 patients (59.2%).

JOA assessments were performed by trained spine surgeons according to standardized institutional procedures to minimize inter-rater variability.

As a secondary outcome, the duration of symptoms prior to surgery was analyzed as a potential predictor of postoperative improvement. Symptom duration was defined as the patient-reported time interval (in months) from the initial onset of myelopathic symptoms (e.g., hand clumsiness, gait disturbance, sensory deficits) to the date of surgical decompression. 

Statistical analysis

All statistical analyses were performed using computer-assisted methods. Statistical analyses were conducted using R (R Foundation for Statistical Computing, Vienna, Austria) (available on the Comprehensive R Archive Network (CRAN) at https://cran.r-project.org) and Microsoft Excel (Microsoft Corp., Redmond, WA, USA). 

Continuous variables are presented as mean ± standard deviation (SD) or median (interquartile range (IQR)) depending on data distribution and categorical variables as counts and percentages (N (%)). The Shapiro-Wilk test was used to assess normality. Non-parametric data were compared using the Wilcoxon signed-rank test. For regression analyses, both linear and logistic multivariate models were constructed. Regression coefficients (β), 95 % confidence intervals (CI), and corresponding p-values are reported. Statistical significance was defined as two-tailed p < 0.05. Test statistics are indicated in table legends where applicable.

Multicollinearity among covariates was assessed by calculating variance inflation factors (VIFs) with values below 5 indicating no significant collinearity.

Twelve-month analyses were conducted as complete-case analyses; no imputation procedures were applied.

## Results

Patient demographics and baseline characteristics

The mean age across the cohort was 66.2 ± 9.5 years, and 89 patients (62.7%) were male. The average preoperative JOA score was 13.1 ± 2.3, and the mean duration of symptoms before surgery was 17.8 months (range: 0.1-60 months). Most patients (n = 76; 53.5%) were classified as ASA II, followed by ASA III (n = 48; 33.8%) and ASA I (n = 18; 12.7%). The mean BMI was 27.5 ± 4.3 kg/m².

Among the 142 patients included in the analysis, 34 patients (23.9%) were classified as having severe myelopathy, 67 patients (47.2%) as moderate, and 41 patients (28.9%) as mild.

Postoperative neurological recovery

Our findings showed continuous postoperative improvement in neurological function as measured by the JOA score. At the three-month follow-up, the mean JOA score increased significantly to 14.42 (±2.1), corresponding to a gain of 1.16 points (8.9%; p < 0.001). 

This functional improvement persisted through the 12-month follow-up, with a mean JOA score of 14.7 (±2.2) and a total increase of 1.6 points (12.2%; p < 0.001), underscoring the durability of the surgical benefit. 

Importantly, the magnitude of change approaches the minimal clinically important difference (MCID) for the JOA score, which is estimated to lie between 1.5 and 2.0 points. Accordingly, the observed improvements are not only statistically significant but also clinically relevant.

When stratified according to preoperative severity, the degree of neurological improvement differed substantially between groups. Patients with severe myelopathy (JOA < 12) showed the greatest functional gains, with a mean improvement of 1.99 points at three months (p < 0.001) and 4.58 points at 12 months (p = 0.0022). In contrast, patients with moderate myelopathy (JOA 12-14.9) improved by 1.03 points at three months (p < 0.001) and 1.24 points at 12 months (p = 0.0034). Those with mild preoperative symptoms (JOA ≥ 15) demonstrated only marginal short-term gains (+0.67 points; p < 0.001), and a slight, but statistically insignificant, decline in function was observed at 12 months (−0.77 points; p = 0.1428). A detailed summary of JOA score changes across severity groups is presented in Table 1.

**Table 1:** JOA score improvement stratified by preoperative severity JOA score improvement at three and 12 months stratified by preoperative severity of cervical myelopathy. Patients were classified into mild (JOA ≥ 15), moderate (JOA 12-14.9), and severe (JOA < 12) groups based on their preoperative JOA scores. The table shows the mean change in JOA score (±standard deviation) at three and 12 months postoperatively. Within-group comparisons were performed using the Wilcoxon signed-rank test, yielding Z = 3.4 (p < 0.001) for mild, Z = 3.7 (p < 0.001) for moderate, and Z = 4.2 (p < 0.001) for severe cases at three months. At 12 months, corresponding test statistics were Z = 0.7 (p = 0.14) for mild, Z = 2.9 (p = 0.003) for moderate, and Z = 3.1 (p = 0.002) for severe cases. Statistical significance was defined as p < 0.05. JOA: Japanese Orthopaedic Association; SD: standard deviation

Severity group	Mean ΔJOA ± SD (3 months)	P-value	Mean ΔJOA ± SD (12 months)	P-value
Mild	+0.67 ± 0.54	<0.001	-0.77 ± 2.19	0.143
Moderate	+1.03 ± 1.08	<0.001	+1.23 ± 2.44	0.003
Severe	+1.99 ± 1.94	<0.001	+4.57 ± 2.56	0.002

These findings suggest that patients with more advanced neurological impairment experience the most pronounced and sustained benefit from surgical decompression, whereas those with mild preoperative symptoms may experience limited or no measurable functional improvement, potentially due to ceiling effects inherent in the JOA scoring system.

Influence of symptom duration on postoperative neurological recovery

Two multivariate linear regression models were constructed to identify predictors of postoperative improvement in JOA score at three and 12 months, respectively. The models included age, sex, BMI, ASA classification, duration of symptoms, and preoperative JOA score as independent variables. Regression coefficients, CI, and significance levels are summarized in Table 2. 

**Table 2:** Multivariate analysis of independent predictors of neurological recovery at three and 12 months Multivariate linear regression analysis of JOA score improvement at three and 12 months postoperatively. Values represent regression coefficients (β) and p-values. Statistical significance is defined as p < 0.05. Negative β values indicate that lower baseline values (e.g., JOA score, ASA class) are associated with greater improvement. Significant predictors at three months included preoperative JOA score (p < 0.001); at 12 months, BMI, ASA classification, and preoperative JOA score were significant (p < 0.05). Model significance (ANOVA F-test): three-month model (F(8,133) = 6.1; p < 0.001) and 12-month model (F(8,75) = 4.7; p < 0.001). BMI: body mass index; ASA: American Society of Anesthesiologists; JOA: Japanese Orthopaedic Association; ANOVA: analysis of variance

	β coefficient (3 months)	P-value (3 months)	β coefficient (12 months)	P-value (12 months)
Age (years)	+0.0094	0.38	+0.012	0.29
Sex (1 = male)	-0.0041	0.98	-0.36	0.12
BMI (kg/m²)	+0.026	0.183	+0.055	0.027
ASA status (1-4)	-0.2756	0.184	-0.631	0.017
Symptom duration (months)	-0.0124	0.189	-0.0007	0.941
Preoperative JOA score (0-17)	-0.2380	<0.001	-0.11	0.048
Surgical approach (anterior/posterior; 1 = anterior)	+0.433	0.069	-0.277	0.30
Number of stenotic segments	+0.041	0.75	+0.10	0.49

VIFs were calculated for all covariates and ranged from 1.07 to 1.41. Since all values are well below the usual threshold of 5, there is no indication of problematic multicollinearity among the predictors.

The three-month model showed preoperative JOA score emerging as the only independent predictor of improvement. Patients with lower baseline function experienced significantly greater postoperative recovery.

In the 12-month model, in addition to the preoperative JOA score, ASA classification and BMI were significantly associated with outcome. Higher ASA scores predicted less improvement, while higher BMI was modestly associated with greater functional gains. Symptom duration was not a significant predictor at both time points, indicating no clear relationship between the timing of surgery and neurological recovery.

Influence of symptom duration on clinically relevant postoperative neurological recovery 

In the multivariate logistic regression analysis (Table 3), we evaluated the likelihood of achieving a clinically relevant neurological recovery (≥1.5-point gain in JOA score) at three and 12 months postoperatively.

**Table 3:** Predictors of clinically meaningful neurological improvement at three and 12 months Multivariate logistic regression analysis of factors associated with achieving a clinically relevant neurological recovery (≥1.5-point gain in JOA score) at three and 12 months postoperatively. Data are presented as adjusted odds ratios with corresponding 95% confidence intervals and p-values. The dependent variable was dichotomized JOA improvement (1 = ≥1.5-point gain; 0 = <1.5-point gain). Covariates entered into both models were age (years), sex (male vs. female), BMI (kg/m²), ASA classification (I-IV), duration of preoperative symptoms (months), preoperative JOA score (0-17 points), surgical approach (anterior vs. posterior), and number of stenotic segments. Overall model (likelihood-ratio χ²): three-month model (χ²(8) = 24.6; p = 0.0016) and 12-month model (χ²(8) = 16.9; p = 0.031). BMI: body mass index; ASA: American Society of Anesthesiologists; JOA: Japanese Orthopaedic Association

	Odds ratio (confidence interval) at 3 months	P-value	Odds ratio (confidence interval) at 12 months	P-value
Age (years)	1.005 (0.99-1.01)	0.249	1.005 (0.99-1.02)	0.309
Sex (1 = male)	1.015 (0.87-1.19)	0.852	0.807 (0.66-0.99)	0.046
BMI (kg/m²)	1.00 (0.99-1.01)	0.948	1.008 (0.98-1.039)	0.451
ASA status (1-4)	0.963 (0.83-1.12)	0.633	0.89 (0.71-1.12)	0.321
Symptom duration (months)	0.993 (0.99-1.00)	0.045	0.998 (0.99-1.01)	0.717
Preoperative JOA score (0-17)	0.935 (0.99-1.00)	<0.001	0.985 (0.94-1.04)	0.570
Surgical approach (anterior/posterior; 1 = anterior)	1.103 (0.93-1.31)	0.276	0.814 (0.64-1.03)	0.095
Number of stenotic segments	0.940 (0.85-1.04)	0.211	1.011 (0.89-1.15)	0.872

The three-month logistic model identified two independent predictors of achieving a clinically relevant JOA score gain (≥1.5 points). Each additional month of preoperative symptoms reduced the odds of meaningful early improvement by about 0.7% (p = 0.045). At the same time, patients who started with a higher baseline JOA score were less likely to reach that threshold, a reflection of the ceiling effect, with each point of higher preoperative function lowering the odds by roughly 6.5% (p < 0.001). None of the other covariates showed a significant association with early recovery.

By 12 months after surgery, the pattern had shifted: only patient sex remained a significant predictor, with men experiencing about a 19.3% lower chance of achieving the ≥1.5-point gain compared to women (p = 0.046). Neither symptom duration (p = 0.717) nor preoperative JOA score (p = 0.570) retained significance, and the remaining factors also showed no independent effect on long-term odds of clinically relevant neurological improvement.

## Discussion

In this study, we evaluated predictors of postoperative neurological improvement in patients with DCM undergoing decompression surgery, focusing on the role of symptom duration. 

Numerous investigations have demonstrated that a shorter interval between symptom onset and surgery, typically less than 4-12 months, is associated with superior functional outcomes, as measured by JOA scores and gait recovery rates. For instance, patients operated on within four months of symptom onset achieve significantly higher JOA scores at one year than those with longer-standing symptoms [5], and symptom durations exceeding 12 months independently predict poorer gait recovery [4,6,10]. Conversely, some reports suggest that, while longer symptom duration is linked to worse preoperative status, the absolute gain from baseline may not differ substantially, indicating that meaningful improvement remains possible even after delayed intervention [6]. Additional variables, such as older age, comorbidities, and radiological markers like T2 hyperintensity, also modulate surgical outcomes, yet early decompression consistently stands out as a modifiable prognostic factor [3].

Our data reaffirm that patients with more severe preoperative myelopathy tend to show the largest absolute gains in JOA scores, likely reflecting greater capacity for measurable recovery from a lower functional baseline and the ceiling effects inherent in the scoring system. In multivariate analysis, baseline JOA score was the sole variable associated with improvement at three months and remained significant at 12 months alongside ASA classification and BMI. Higher ASA status predicted attenuated recovery, underscoring the influence of systemic health, while a higher BMI was unexpectedly associated with marginally greater functional gains; this association is likely influenced by unmeasured factors (e.g., muscle mass or selection patterns) and should therefore be interpreted cautiously.

Although symptom duration did not independently influence continuous JOA change, logistic regression showed a small reduction in the likelihood of early clinically meaningful improvement (OR: 0.993; p = 0.045). By 12 months, however, neither symptom duration nor most other covariates remained significant, while patient sex continued to predict clinically relevant improvement, with men less likely than women to achieve the MCID threshold. The reason for this gender disparity is unclear, but may involve differences in health-seeking behavior, hormonal influences on neural repair, or other sociocultural factors. Our results support the notion that, although substantial recovery can occur even after protracted symptom duration, earlier surgical intervention maximizes the likelihood of rapid and clinically meaningful improvement. In practice, this underscores the importance of prompt recognition and referral of patients with DCM, particularly those presenting with moderate or severe deficits. Moreover, optimization of comorbidities (reflected in ASA status) before surgery and attention to nutritional status or body composition may further enhance outcomes.

This study has several limitations that should be considered when interpreting the findings. First, its retrospective single-center design limits control over confounding variables and reduces generalizability, as noted by both reviewers. Second, symptom duration was based on patient self-report and is therefore susceptible to recall bias, which may have introduced measurement inaccuracies. Third, although the JOA score is an established and widely used clinical metric, its ordinal structure and ceiling effects, particularly in patients with mild myelopathy, may have reduced sensitivity to subtle postoperative changes. Furthermore, despite standardized institutional procedures and trained assessors, inter-rater variability in JOA scoring cannot be fully excluded. A further limitation concerns the incomplete 12-month follow-up: only 59% of patients had long-term data available. This attrition may have introduced bias, with the potential to either overestimate or underestimate true long-term recovery patterns. Sensitivity analyses to account for missing data were considered but were not feasible due to the reduced sample size at the final time point; thus, all 12-month outcomes were analyzed using a complete-case approach. Finally, although we attempted to corroborate reported symptom duration with available clinical documentation, discrepancies remain possible. Future prospective multicenter studies with standardized symptom-onset documentation, complementary patient-reported outcomes, and more complete follow-up would help to validate and extend these findings.

## Conclusions

Our analysis demonstrates that the degree of preoperative neurological impairment is the most powerful predictor of the magnitude of postoperative recovery in patients undergoing surgical decompression for DCM. While earlier surgery modestly increases the likelihood of achieving clinically meaningful improvement within the first few months, meaningful gains remain attainable even after longer symptom durations. Systemic health, as reflected by ASA classification, further influences long‐term outcomes.
